# Potential Protective Role Exerted by Secoiridoids from *Olea europaea* L. in Cancer, Cardiovascular, Neurodegenerative, Aging-Related, and Immunoinflammatory Diseases

**DOI:** 10.3390/antiox9020149

**Published:** 2020-02-10

**Authors:** María Luisa Castejón, Tatiana Montoya, Catalina Alarcón-de-la-Lastra, Marina Sánchez-Hidalgo

**Affiliations:** Department of Pharmacology, School of Pharmacy, University of Seville, 41012 Sevilla, Spain; mcastejon1@us.es (M.L.C.); tmontoya@us.es (T.M.); calarcon@us.es (C.A.-d.-l.-L.)

**Keywords:** immunomodulation, inflammation, olive tree, oxidative stress, secoirioids

## Abstract

Iridoids, which have beneficial health properties, include a wide group of cyclopentane [*c*] pyran monoterpenoids present in plants and insects. The cleavage of the cyclopentane ring leads to secoiridoids. Mainly, secoiridoids have shown a variety of pharmacological effects including anti-diabetic, antioxidant, anti-inflammatory, immunosuppressive, neuroprotective, anti-cancer, and anti-obesity, which increase the interest of studying these types of bioactive compounds in depth. Secoiridoids are thoroughly distributed in several families of plants such as Oleaceae, Valerianaceae, Gentianaceae and Pedialaceae, among others. Specifically, *Olea europaea* L. (Oleaceae) is rich in oleuropein (OL), dimethyl-OL, and ligstroside secoiridoids, and their hydrolysis derivatives are mostly OL-aglycone, oleocanthal (OLE), oleacein (OLA), elenolate, oleoside-11-methyl ester, elenoic acid, hydroxytyrosol (HTy), and tyrosol (Ty). These compounds have proved their efficacy in the management of diabetes, cardiovascular and neurodegenerative disorders, cancer, and viral and microbial infections. Particularly, the antioxidant, anti-inflammatory, and immunomodulatory properties of secoiridoids from the olive tree (*Olea europaea* L. (Oleaceae)) have been suggested as a potential application in a large number of inflammatory and reactive oxygen species (ROS)-mediated diseases. Thus, the purpose of this review is to summarize recent advances in the protective role of secoiridoids derived from the olive tree (preclinical studies and clinical trials) in diseases with an important pathogenic contribution of oxidative and peroxidative stress and damage, focusing on their plausible mechanisms of the action involved.

## 1. Introduction

Iridoids, which have beneficial health properties, include a wide group of cyclopentane [*c*] pyran monoterpenoids present in plants and insects. The name iridoid derived from *Iridomyrmex*, a genus of fornices from which iridomirmecin and iridodial compounds were isolated. These products have been considered as defensive compounds. In fact, the biosynthesis of these derivatives of monoterpenes takes place in the different organisms by similar pathways; defense is its main role, and in the case of insects, they are used as sex pheromones [1].

Iridoids were first isolated in the latter part of the 19th century, but Halpern and Schmid proposed the basic skeleton of the iridoids in their investigation of the structure of plumieride in 1958 [2]. Particularly, they are secondary metabolites of terrestrial and marine flora and fauna, being found in a large number of plants families, usually as glycosides. For this reason, some of them are chemotaxonomically useful as markers of genus in various plant families. Besides, they exhibit a wide range of bioactivities including anti-inflammatory, antibacterial, anti-carcinogenic, and antiviral activities [3]. In fact, they are used as bitter tonics, sedatives, antipyretics, cough drugs, remedies for skin disorders, and as hypotensive agents. In addition, they are useful as an antidote in mushroom intoxications by *Amanita* type.

The cleavage of the cyclopentane ring of iridoids leads to secoiridoids. Mainly, secoiridoids have shown a large variety of pharmacological properties including anti-diabetic, anti-inflammatory, immunosuppressive, neuroprotective, anti-cancer, and anti-obesity. This fact encouraged us to study the bioactivities of these phytochemicals in depth and update the latest preclinical and clinical data on its bioactivity and potential therapeutic uses.

### 1.1. Structure and Classification

Several classifications have been developed over the years, given the variety and complexity of iridoids and secoiridoids [4,5].

From 1980 to date, bibliographic data has used the classification proposed by El-Naggar and Beal [2], who categorize these compounds according to the number of carbons included in their structure:

- Group 1: C_8_ iridoids (di-nor-iridoids)
- Group 2: C_9_ iridoids (nor-iridoids)
- Group 3: C_10_ iridoids, which occur mainly as glycosides
- Group 4: Aglycones and some iridoids included in the other three groups lacking a sugar residue in their structure
- Group 5: Iridoids derivatives. This group comprises compounds derived from the opening of the pyran ring
- Group 6: Included bis-iridoids as a result of condensation of two monomers, (a) directly as in iridolinalin A, or (b) through a sugar residue as in globuloside A.

At the same time, there are other classifications of secoiridoids according to the presence of these compounds in certain families, including the Oleaceae family. In fact, a total of 232 secoiridoids (aglycones, glycosides, derivatives, and dimers) have been isolated from nine genus of the family Oleaceae. These genera include Fontanesia, Fraxinus, Jasminum, Ligustrum, Olea, Osmanthus, Phillyrea, Picconia, and Syringa, and these secoiridoids were classified into other five groups [6]:

- Simple secoiridoids: Generally, for the simple secoiridoids, positions C_7_ and C_11_ have either a free carboxylic acid group or a methyl ethyl ester derivative of the acid. The configurations of the positions C_1_ and C_5_ are *S*.
- Conjugated secoiridoids: This group of compounds is the most numerous secoiridoids isolated from the Oleaceae family. The name of the class derives from the type of compound that is linked or conjugated to the secoiridoid nucleus. Based on this, this class is further categorized into seven subgroups: aromatic-conjugated, sugar-conjugated, terpene-conjugated, cyclopentane-conjugated, coumarin-conjugated, lignans-conjugated, and other secoiridoids. Normally, the conjugations occur in C_7_ due to this position, which is is usually oxidized to a carboxylic acid and esterified with diverse groups.
- 10-Oxyderivative of oleoside secoiridoids: This group contains the oleoside nucleus with distinct structural differences. The C_8_ and C_9_ positions exist as double bonds, with a hydroxy group at the C_8_ position or an ester formed by an oxygen atom with different groups. A total of 40 10-Oxyderivative of oleoside secoiridoids have been isolated from the Oleaceae family.
- *Z*-Secoiridoids: This class of secoiridoids is characterized by the presence of double-bond geometry at the C_8_ in *Z*-configuration; however, only five compounds have been isolated from the Oleaceae family.
- Secologanosides and oxidized secologanoside secoiridoids: Compounds of this class are based on the secologanoside nucleus. They are differentiated by the positions on the C–C double bond between C_8_ and C_10_ and C_10_ oxidation level.

### 1.2. Main Naturally Occurring Iridoids and Secoiridoids Present in Olea europaea L

Iridoids and secoriridoids are thoroughly distributed in the plants of class Magnoliopside, concretely belonging to the following families: *Scropulariaceae, Verbenaceae, Lamiaceae, Apocynaceae, Loganiaceae, Bignoniaceae, Plantaginaceae, Rubiaceae, Pedaliaceae, Cornaceae, Acantheceae, Loasaceae, Lentibulariaceae, Gentianaceae, Oleaceae, Nyctanthaceae, Caprifoliaceae, Dispsacaceae,* and *Valerianaceae*.

For instance, *Valeriana officinalis* L. (Valerianaceae), *Harpagophytum procumbens* L. (Pedaliaceae), *Genciana lutea* L. (Gentianaceae), *Fraxinus excelsior* L. (Oleaceae) and *Olea europaea* L. (Oleaceae) are the most representative medicinal plants commonly used in medicine, due to their iridod/secoiridoid content [3]. Particularly, *Olea europaea* L. (Oleaceae) is a small evergreen tree with firm branches and a grayish bark. The leaves are lanceolate, opposite, short-petioled, mucronate, green above and hoary on the underside. On the other hand, the flowers are small, short, erect racemes, axillary, very much shorter than the leaves, and the fruit is a small smooth, purple, or green drup, with a nauseous, bitter flesh, enclosing a sharp-pointed stone [7]. 

*Olea europaea* L. preparations have been traditional used in folk medicine in the European Mediterranean area, Arabia peninsula, India and other tropical and subtropical regions, as a diuretic, emollient, hypotensive, and for urinary and bladder infections [8]. Most of the plant parts of *Olea europaea* L. are used in the traditional system of medicine around the world. Oil is taken with lemon juice to treat gall stones [9]. Leaves are taken orally for stomach and intestinal diseases and used as mouth cleanser [10], and the decoction of dried leaves is taken orally for diabetes [11]. An extract of the fresh leaves is taken orally to treat hypertension and to induce diuresis [12], whereas an infusion of the fresh leaves is taken orally as an alternative treatment for inflammatory diseases [13]. Similarly, essential oil extracted from the fruit is also used to treat rheumatism, promote blood circulation [14], and as a laxative [15].

**Figure 1:**
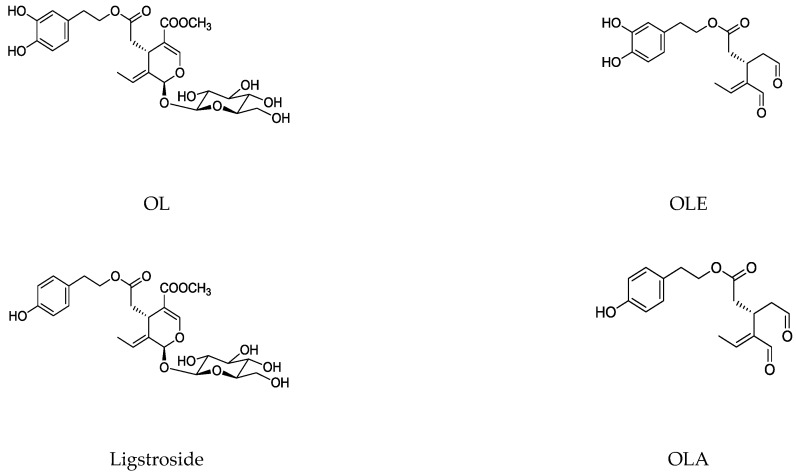
Chemical structures of secoiridoids most abundant in olive trees. OL: oleuropein, OLA: oleacein, OLE: oleocanthal.

The main biophenol secoiridoids found in the olive tree include: oleuropein (OL), dimethyl-OL, ligstroside, and their hydrolysis derivatives such as OL-aglycone, oleocanthal (OLE), oleacein (OLA), elenolate, oleoside-11-methyl ester, elenoic acid, hydroxytyrosol (HTy), and tyrosol (Ty) (Figure 1). 

### 1.3. Biosynthesis and Biotransformation of Secoiridoids in Olive Tree

The amount and distribution of secoiridoids present in olive tissues depend on various environmental factors such as the ripening cycle, geographical origin, and cultivation practices, among others. Besides, the content of phenolic glycosides as patterns and the activity of endogenous enzymes can play a role in the quantitative composition of secoiridoids in the olive tree [16].

The fact that secoiridoids are mostly present in early stages is due to the enzymatic and chemistry reactions that take place in the maturation time. In this sense, three different states in fruit maturation have been described: growth phase, green maturation phase, and black maturation phase, which is characterized by the presence of anthocyans. 

OL is mainly abundant in early stages, although its levels decrease during the maturation process. In fact, OL decreases quickly in black crops and is not present in some varieties of Oleaceae.

**Figure 2:**
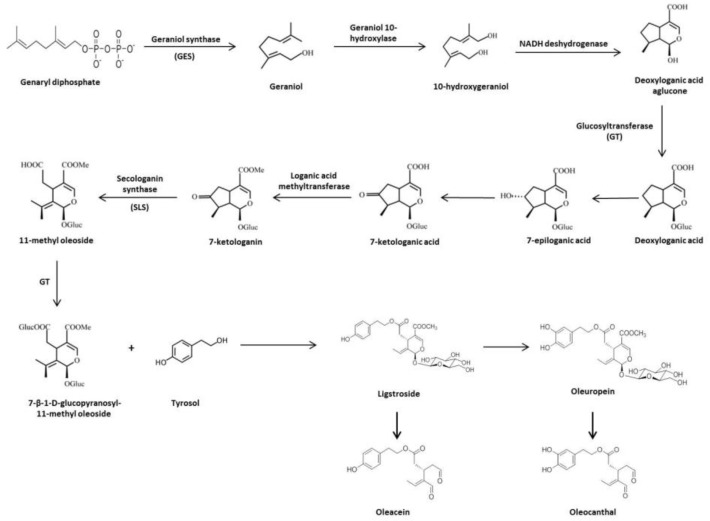
Biosynthesis and biotransformation of secoiridoids in *Olea europaea* L.

The main precursor of OL and ligstroside is oleoside 11-methyl ester (elenolic acid glucoside). Firstly, geraniol synthase (GES) catalyzes the transformation of genaryl diphosphate to geraniol, which is converted to 10-hydroxygeraniol by the geraniol 10-hydroxylase enzyme. The iridioids in Oleaceae must be formed from this point with 10-hydroxygeraniol as the starting compound via irididal and iridotrial up to deoxyloganic acid, which is the precursor of loganin and loganic acid, as well as secologanin and secologanic acid [17]. From this point, up to five routes have been proposed to explain the origin of all iridoids found in this family. However, it is known that most of the secoiridoids present in *Olea europaea L.* are derived from deoxyloganic acid as a common intermediate [17,18]. Following this line, nicotinamide adenine dinucleotide deshydrogenase (NADH) acts on 10-hydroxygeraniol to form deoxyloganic acid aglucone. The transfer of glucosyl groups to deoxyloganic acid aglucone (precursor of monoterpene indolic alcaloids and OL) is catalyzed by glucosyltransferase (GT). Deoxyloganic acid experiments a 7-α-hydroxylation of the cyclopentane ring and forms 7-epiloganic acid, which quickly goes to 7-ketologanic acid through hydroxyl group oxidation. Loganic acid methyltransferase catalyzes 7-ketologanin syntheses. In this point, secologanin synthase (SLS) oxides a ketonic group to form 11-methyl oleoside, which is immediately glucosylated by GT. Finally, 7-β-1-D-glucopyranosyl-11-methyl oleoside is esterified with Ty to produce ligstroside, and then OL is formed [17,19] (Figure 2). 

Secoiridoids are distributed throughout the tissues of the olive tree, but their nature and concentration change among different parts of the plant. Thus, biosynthetic or mechanicals transformation during production are decisive to quantify alterations of the bioactive small molecules [18]. Particularly, OL is the major secoiridoid constituent of unripe drupes (peel, pulp, and seed). The amount of OL decreases along fruit maturation, as commented above, whereas its aglycon form increases its levels. OL-aglycone is formed by the cleavage of the glycosidic bond mediated by β-glucosidase activity. Ligstroside has been described as a common phenolic component in different olive tissues (leaf, fruit pulp, and stone) and olive oil, but it has been rarely found in olive seeds [20].

In the course of maturation, OL and ligstroside are considered pattern components. Both of them are present in the olive fruit, but they are almost non-existent in olive oil (85–95% reduction) [21,22]. β-glucosidase acts by decreasing OL and ligstroside levels, aglycon forms from OL, and ligstroside can be detected as isomers due to the keto-enolic tautomeric equilibrium of the elenolic acid moiety [23,24]. 

Other dialdehydics structurally related to these secoiridoid precursors are OLA and OLE. Different authors have reported that both OLA and OLE levels increase during ripening due to OL and ligstroside degradation, respectively [25]. Thus, they concluded that OL and ligstroside are natural precursors of OLA and OLE as breakdown products resulting from enzymatic activity during the extraction and maturation processes [26,27,28,29].

OL and ligstroside have been detected in olive leaves. In turn, OLA and OLE levels are augmented in mature fruits, such as OL and ligstroside aglycons. Moreover, OLA and OL-aglycone are more plentiful in olive oil [16].

### 1.4. Functional and Physiological Chemistry of the Main Secoiridoids of Potential Medical Interest

ROS have a remarkable role in the development of oxidative stress and also in the pathology of numerous diseases. For example, oxidative stress is one of the major cellular features in the onset of many pathological conditions such as Alzheimer’s and Parkinson disease, renal injury, diabetes, cardiovascular diseases, cancer and aging; it occurs when excessive ROS accumulation produced during the normal cell metabolic processes is unbalanced by the antioxidant defence system [30], and it may induce the oxidative modification of cellular macromolecules including lipids, proteins, and nucleic acids [31]. 

Previous epidemiological studies show that the Mediterranean diet is associated with a low incidence of cardiovascular disease or cancer. This may reflect the nutritional effects of the bioactive compounds contained in its major source of fatty acids, i.e., extra-virgin olive oil (EVOO), which is rich in phenolic compounds [32,33]. The phenolic compounds contained in EVOO include HTy, Ty, and their secoiridoids precursors such as OL, OLE, or OLA, among others.

A large number of studies highlighted the antioxidant properties of these compounds including their abilities to promote the activity of ROS-detoxifying enzymes, such as superoxide dismutase (SOD), catalase (CAT), glutathione reductase (GSR) and glutathione S-transferase (GST), to compete with coenzyme Q as an electron carrier in the mitochondrial electron transport chain, which is a site of ROS generation, to act as free-radical-scavenging antioxidants and to inhibit lipid peroxidation [31,34]. In particular, it was shown that dietary OL and OLE treatments were associated with reductions in the production of superoxide anion radical in human monocytes from healthy donors [35]. Similarly, OL administration significantly increased the SOD and glutathione peroxidase (GPx) activity levels in the obstructed kidneys from rats with unilateral ureteral obstruction induced-kidney injury [36]. These results are in accordance with other reports showing that OL was able to increase the amount of enzymes such as GPx and SOD in gentamicin-induced renal toxicity and cisplatin-induced renal injury models [37,38]. The phenolic compounds present in *Olea europaea* L. activated enzymatic and non-enzymatic antioxidant defense mechanisms, particularly preventing cell membrane damage by high-dose UV-B rays [39]. 

Vitamin E (α-tocopherol) is an essential micronutrient in the diet of all mammals, and it is a potent antioxidant in biological systems. This compound is the main chain-breaking antioxidant that prevents the propagation of free radicals reactions, and consequently prevents the tissue damage. In this sense, it has been reported that supplementing the diet with OL for 21 days maintained higher levels of α-tocopherol in liver of female Wistar rats [40]. 

The most important antioxidant activity of the olive tree phenolic compounds is related to the free-scavenging ability because they inhibit the propagation chain during the oxidation process through the donation of radical hydrogen to alkylproxyl radicals and the formation of stable derivatives during this reaction. These compounds also act as metal chelators, preventing the generation of high concentrations of hydroxyl radicals [41]. This capacity may be reduced by the presence of the –COOOH_3_ fragment in several secoirioids structures, because it seems to cause a decrease in the antioxidant activity which is related to the inability of this group to act as an H-donor [42]. In fact, OL-aglycone presents better radical-scavenging capacity than single hydroxyl substitutions, such as Ty, and also protects low-density lipoprotein (LDL) from oxidation [43].

The relationship between oxidative stress and inflammation has been established by many authors. The pathogenic role of mixed advanced glycoxidation products (AGE) and advanced lipid peroxidation products (ALE) generated in the course of oxidative stress and their adducts with cell biomolecules, such as proteins and nucleic acids, in a number of chronic inflammatory and autoimmune diseases is well documented [44].

On the other hand, in the past years, the leaves of *Olea europaea* L. have been considered as an important source of antioxidant compounds. In this case, OL is the most predominant and active phenolic compound (60–90 mg/g dried olive leaves), and it is usually considered as an antioxidant reference in comparison with other secoirioids [45,46]. OL contains active components in its molecule with conferred a potential antioxidant activity. It has been suggested that these properties are related to the H-atom donation from the phenolic groups present in OL. For example, OL administration showed a protective effect against ROS production in endothelial cells since OL showed good cytocompatibility and antioxidant activity, which revealed effectiveness in controlling the oxidative stress upon exposure to H_2_O_2_ [47]. In addition, OL presented slightly weaker radical scavenging activity than HTy by 2,7-dichlorodihydrofluorescein diacetate (DCFH-DA) and ABTS methods [48] and the ability to inhibit LDL oxidation by scavenging free radicals [49]. 

Although the studies about the antioxidant activity of OLE are limited, it has been demonstrated that OLE could inhibit nicotinamide adenine dinucleotide phosphate oxidase (NOX) in isolated human monocytes, and also OLE was able to reduce intracellular ROS levels in SH-SY5Y cells. Recently, Montoya et al. have also showed that OLE produced a potent reduction of intracellular ROS and nitrites production in LPS-induced murine peritoneal macrophages [50]. 

OLA is one of the major phenolic compounds present in the olive tree, but its antioxidant profile is more unknown in comparison with other secoirioids, such as OL. However, OLA has demonstrated to be a potent scavenger of HOCl and myeloperoxidase release and exerts a stronger inhibitory capacity of neutrophil’s oxidative in comparison to OL [32].

### 1.5. Pharmacokinetics of Secoiridoids from the Olive Tree

Numerous investigations have revelead the beneficial effects of olive leaves and olive oil for the treatment of many diseases. The possibility that their constituents may achieve any biological effects depends on the chance of reaching molecular targets in a specific tissue or organ at a sufficient dose, which is dependent on their metabolism and bioavailability [51].

The mechanism of intestinal absorption of olive oil phenols is unclear, as it does not appear to be strictly dependent on the polarity of these compounds. For example, OL, which is apolar, is able to diffuse through the lipid bilayer of the epithelial cell membrane. However, also, the more polar HTy appears to be absorbed via a passive bidirectional diffusion mechanism [52,53].

To date, reports regarding the bioavailability of the phenolic compounds present in olive oil are extended, whereas bioavailability studies concerning secoiridoids derivatives such as OL, OLE, and OLA have been scarcely studied. Thus, data on the bioavailability of these secoiridoids compounds in human and even animals would be of great interest in order to establish their potential health benefits.

Generally, secoiridoids from *Olea Europaea L.* are mainly present in glycosylated forms (OL and ligstroside). For this reason, after oral administration, enzyme from saliva starts a hydrolysis process continued in the stomach by digestive enzymes and β-glycosidases. The unmodified forms could be absorbed to the small intestine or colon, where they are hydrolyzed, finally [54]. The result of this hydrolytic process is the formation of derived aglycon forms. At the same time, the aglycons that were formed could be absorbed in the small intestine or colon. The chemical structure and vehicle of administration are decisive for the rate and extension of gastrointestinal absorption. Structural modifications occur via conjugation in small intestinal epithelial cells or in liver, after transport through the portal system. Metabolites reach the general blood circle, from which they are excreted in the urine [55].

Related to OL, some authors suggest that it could be absorbed in the small intestine or colon. In this sense, Kendall et al. reported that OL diffuses in the stomach and remained stable and intact at the gastric level during digestion being absorbed in the small intestine in healthy young adults [56]. Similarly, several studies have established that OL is stable at the gastric level during digestion, since the bioavailability of its main metabolite HTy is higher [57]. On the contrary, Corona et al. determined that OL was absorbed in the colon and degraded by gut microbiota in rat intestinal segment that produces HTy, which expresses biological activity [58]. Further studies developed in rats confirmed that OL and HTy were present in plasma, feces, and urine as such, and also conjugated as glucuronide after oral OL administration [59,60,61]. Besides, there are several studies that focus on the study of the colonic pathway of phenolic compounds present in the olive tree. For example, Mosele et al. have demonstrated that OL hydrolysis formed OL-aglycone, elenoic acid, HTy and HTy-Ac in vitro, which is probably a consequence of the hydrolytic transformation making all of them less resistant to the gastric acidic hydrolysis in comparison to OL and more sensitive to temperature, pH, and enzyme activity. However, OL administration detected other related metabolites such as homovanillic acid in rats [62]. These divergences could be explained taking into account the differences found between both murine and human models. 

Surprisingly, it has been postulated that plasma peak at 1 hour (h) after OL administration for humans or 2 h for a murine model, although OL could be detected after 10 minutes (min) [56,63]. In addition, glucuronides and OL sulfate derivatives could be detected in plasma (at 23 min) or in the urine (at 8 h) after ingestion. Particularly, De Bock and colleagues described a heterogeneous effect in OL leaf extract bioavailability and metabolism that was dependent on a number of factors, including preparation (capsule/liquid) and gender [63]. In fact, they found that compared to capsules, OL leaf extract in a liquid formulation led to a greater OL peak levels and area under the curve (AUC) in plasma and described that males may be more efficient at conjugating OL. 

The production of metabolites during the metabolism process of these compounds could be interesting given that these new compounds may also exert beneficial effects on the organism. The metabolism of secoiridoids can be carried out by phase I (hydrogeneration, hydroxylation, hydratation, etc) or phase II reactions (glucuronidation, methylation, sulfation, etc.). A perfused rat intestinal model determined that the major small intestine metabolites from OL-aglycone were glucoronide conjugates, so they are not absorbed in parental form [64]. OL-aglycone and ligstroside aglycone present a 55%–66% rate of absorption in humans. The aglycon forms were excreted in urine as HTy or Ty [65]. Particularly, during gastric digestion, OL, OL-aglycone, and other phenolic compounds are transformed into HTy, which is throughout transformed into its phase II metabolites (glucuronide and sulfate conjugates) and into HTy-Ac by effect of the acetyl-CoA enzyme [66]. OL and ligstroside formed sulfate and glucuronide conjugates during absoption after undergoing extensive first-pass intestinal/hepatic metabolism, whereas concentrations of their free forms are not detected in the body fluids. These compounds were absorbed quickly after oral administration and then were metabolized and excreted in the urine mainly as glucuronide [67]. The catabolism of OL could produce phenylacetic and phenylpropionic catabolites which then could be absorbed and subsequently transforme into phase II metabolites. Thus, compounds such as ligstroside or OL could be absorbed as such or reabsorbed in the form of their respective glucuronide conjugates [58,68].

The bioavilability of OLE is still very limited. Ligstroside aglycone and OLE are hydrolyzed in the acidic gastric environment in the stomach, leaving free Ty after 30 min. OLE is absorbed in the small intestine mediated by passive diffusion through the membrane, which is favorable given an adequate coefficient of partition (log P = 1.02) and stability in acid gastric [69]. In fact, Romero et al. verified that OLE was stable in gastric acid conditions at 37 °C for 4 h [70]. Although it has been postulated that OLE is mainly metabolized by Phase I (hydrogenation, hydroxylation, and hydration) [71,72], some hydrogenated metabolites pass to Phase II of metabolism as glucoronidated forms [54], whereas methylated or sulfate forms of OLE have not been detected in any study in humans to date [71,72]. The report published by García-Villalba et al. revealed that OLE and several secoiridoids metabolites were excreted in human urine between 2 and 6 h after olive oil ingestion [72]. 

On the other hand, OLA may be absorbed in the small intestine by passive diffusion through the membrane due to its favorable partition coafficient. OLA was found to be stable at gastric acid remaing unalterated after 4 h of incubation [73]. After the consumption of olive oil, De las hazas et al. [66] described that OLA was hydrolyzed into HTy and elenoic acid in the digestive system and further metabolized by the enzymatic systems. 

In conclusion, there are a lot of plants that have been used as medicines since time immemorial; specificallys, *Olea europaea* L. is a species rich in compounds that have proven their efficacy in the management of several complex diseases including cardiovascular disorders, diabetes, and viral and microbial infections, but additional works are still really necessary to explore the evidences for other traditional uses of this plant. Particularly, the antioxidant, anti-inflammatory, and immunomodulatory properties of secoiridoids from the olive tree (leaves and fruits) have been suggested as a potential application in several oxidative stress-mediated diseases including cancer, cardiovascular disorders, neurodegeneration, the aging process and immunoinflammatory diseases. Thus, the purpose of this review is summarize recent advances in the protective role of these secoiridoids derived from olive tree (preclinical and clinical studies) in these pathologies derived from oxidative stress and focusing on their plausible mechanisms of action involved.

## 2. Protective Role of the Olive Tree Secoiridois in Diseases with an Important Pathogenic Contribution of Oxidative and Peroxidative Damage

### 2.1. Olive Tree Secoiridoids and Cancer

Cancer is a complex chronic degenerative disease characterized by a multistep process in which normal cells turn into malignant cells, acquiring several properties such as abnormal proliferation and reduced apoptosis.

The main factors that cause the majority of cancer cases are tobacco and dietary habits. In fact, there is an estimation that around 30% of all cancers may be avoidable by changing food intake [74,75]. Therefore, the identification and characterization of foods and their components, which could prevent the incidence and development of cancer, is an important objective for modern nutritional research [74,76]. In this sense, it is important to take into account that populations who are living near to the Mediterranean area have a lower incidence of cancer compared to other regions. This fact is probably due to the consumption of the diet known as the Mediterranean diet [77]. Besides, it is well-known that the pathophysiology of common diseases states such as cancer, cardiovascular disease, arthritis, and neurodegenerative diseases, among others, are associated with chronic inflammation [78].

There are a large numbers of studies that support the chemopreventive role of natural compounds derived from EVOO and the olive tree such as OL, OLE, OLA, or ligstroside against different cancers and inflammation process. Particularly, the role of secoiridoids derived from *Olea europaea* L. has been investigated in different types of cancerous processes (Table 1 and Table 2). 

OL is the most abundant of the phenolic compounds in olives. It could scavenge reactive oxygen and nitrogen species as well as promote nitric oxide (NO) production in macrophages. In fact, it has been postulated that OL may be the major factor responsible for the beneficial effects of the Mediterranean diet against tumor growth [79]. 

There are several studies describing the potential role of OL in breast cancer. For example, researchers have established the beneficial effects of OL in MCF-7, MCF-10A, and MDA-MB-231 human breast cancer cells where OL was able to decrease the expression of histone deacetylase II (HDAC2), HDAC3, and HDAC4, induce apoptosis, and retard cell migration and invasion in a dose-dependent manner [88,89]. Besides, OL produced tissue inhibitors of metalloproteinases (TIMPs) overexpression and metalloproteinases (MMPs) genes down-regulation, which could help in the prevention of breast cancer metastasis [82]. Similarly, OL treatment reduced MDA-MB-231 cell viability in a dose-dependent manner and significantly suppressed hepatocyte growth factor (HGF) and 3-methyladenine (3-MA) inducing cell migration and invasion [90].

In human HT29 colon adenocarcinoma cell line, OL limited the growth and induced apoptosis via p53 pathway activation, adapting the hypoxia-inducible factor 1-α (HIF-1α) response to hypoxia [81]. In addition, OL induced anti-proliferative and pro-apoptotic effects in a range of doses from 0 to 100 µM in HT29 cells [80]. 

On the other hand, OL induced apoptosis in hepatocellular carcinoma (HCC) via the suppression of the phosphatidylinositol 3-kinase and protein kinase B (PI3K/Akt) pathway [130]. In fact, in combination with other compounds, such as cisplatin, OL could lead to more effective chemotherapeutic combination against HCC [130]. Similarly, in HeLa human cervical carcinoma cells, OL-induced apoptosis was activated by the c-Jun N-terminal kinase (JNK)/Ste20-like proline alanine rich kinase (SPAK) signal pathways [96], and both OL and its peracetylated derivative were able to inhibit thyroid cancer cell proliferation acting on the growth-promoting signal pathway in the TCP-1 and BCPAP thyroid tumor cell lines [95]. Likewise, OL reduced cell viability in human prostate cancer androgen-responsive cells [94], induced autophagy and caused cell cycle arrest in SH-SY5Y human neuroblastoma cells [97,108], and reduced cell viability in U251 and A172 human glioma cancer cells [98]. 

Similarly, there are several in vivo studies that have showed the beneficial effects of OL in different cancer models. For example, Giner et al. have demonstrated that OL prevented the development of colonic neoplasia in dextran sulfate sodium (DSS)-induced colorrectal cancer (CRC) in mice by ameliorating colon inflammatory processes [124]. Besides, there was a relation between the consumption of a high-fat diet (HFD) and the development of solid tumors, which was satisfactorily suppressed with OL-enriched diets reducing the HFD-induced expression of angiogenesis, lymphangiogenesis, and hypoxia markers [126]. Elamin et al. have shown that OL and doxorubicin (DOX) combined treatment down-regulated the antiapoptosis and proliferation protein in a murine model of breast cancer [83]. 

OLE is a bioactive micronutrient in the Mediterranean diet that may be associated with positive findings in some epidemiological studies that suggest a fewer incidences of breast, colon cancer, and other malignancies compared to western and other populations. There are several studies to enhance the beneficial role of OLE in this type of disease (Table 1 and Table 2). In fact, authors believe that consuming more EVOO with high OLE content is a prudent dietary approach to prevent cancer with the caveat that dietary oils convey calories and consequently other caloric sources will have to yield to avoid obesity [131]. OLE is a unique tyrosine-protein kinase Met (c-MET) inhibitor for the control of breast cancer progression and loco regional recurrence [113,114]. The combination of OLE with lapatinib (LP) treatment would allow the use of reduced dose of targeted therapies such as LP, which would reduce future resistance emergence and drug toxicity while maintaining maximal therapeutic activity [115]. Besides, OLE has been able to reduce c-MET kinase activity, cell growth, and the migration and invasion of breast cancer cells; induce G1 cell cycle arrest and apoptosis; as well as inhibit c-MET-dependent signaling in cultured breast cancer cells and tumorigenicity in in vivo murine models [111].

OLE showed the capacity to inhibit breast cancer locoregional recurrence in luminal HER^2+^/ER^+^ BT-474 tumors. The prevention of tumor recurrence was associated with the down-regulation of MET and HER^2^ receptors and suppression of receptor activation [129]. OLE showed a strong anti-proliferative role against several breast cancer cell lines; for example, OLE was able to down-regulate the expression of phosphorylated mammalian target of rapamycin (mTOR) in metastatic MDA-BD-231 breast cancer cell lines [112] and inhibit the proliferation, migration, and invasion of the epithelial human breast cancer and prostate cancer cell lines (MCF7; MDA-BD-231; and PC3). In addition, LeGendre et al. demonstrated that OLE selectively and rapidly induces cell death in cancer cells without being cytotoxic to noncancerous cells. OLE induced cell death by entering the lysosome and inhibiting acid sphingomyelinase (ASM) activity, which induced lyposomal membrane permeabilization (LMP). Even the consumption of 10 mg/Kg oral daily OLE water emulsion treatment significantly suppressed the MDA-BD-231 tumor growth by 90% [132].

Multiple myeloma (MM) is another disease that would be approached with OLE treatment. MM is a plasma cell malignancy that causes devastating bone destruction by activating osteoclasts in the bone marrow milieu. OLE produced the inhibition of macrophage inflammatory 1 alpha (MIP-α) expression and secretion in MM cells proliferation by inducing the activation of apoptosis mechanisms and by down-regulating extracellular signal-regulated kinase (ERK)_1/2_ and protein kinase B (Akt) signal transduction pathways [133]. Besides, OLE was able to inhibit hepatocellular cancer tumor growth and metastasis by preventing signal transducer and activator of transcription (STAT)3 in in vitro and in vivo models. In fact, OLE inhibited proliferation and cell cycle progression in different HCC cell lines, and it also inhibited HCC cells migration and invasion in in vitro models [117].

OLA, also known as 3,4-(dihydrophenyl) ethanol (3,4-DHPEA-EDA) has antioxidant, anti-inflammatory, and anti-microbial activities well documented in previous studies, but its effects on tumor biology are still poorly defined [122]. Particularly, published studies reporting the beneficial properties of OLA in the development of several cancers have been summarized in Table 1 and Table 2. In this sense, OLA was able to reduce the DNA damage in HL60 promyelocytic leukemia cells when co-incubated with H_2_O_2_ in the medium [43] and also presented similar effects to OLE in the reduction of viability and migration of non-melanoma skin cancer cells and in the inhibition of proliferation of ERK and Akt phosphorylation and particularly through the reduction of B-Raf expression [121]. Likewise, OLA reduced the viability of MM primary samples and cell lines even in the presence of bone marrow stromal cells (BMSCs) [84].

### 2.2. Olive Tree Secoiridoids and Cardiovascular Diseases

The Global Burden of Disease indicates that cardiovascular diseases are still the main cause of global death, representing about 31% of total deaths in the world in 2015 [134]. These pathologies affect heart and vessels, as coronary heart disease, cerebrovascular disease, peripheral arterial disease, and pulmonary embolism, among others [135]. There is evidence that suggests a possible link between inflammation, endothelial dysfunction, and cardiovascular diseases are increased by oxidative stress. Oxidative stress plays a critical role in the development and progression of atherosclerosis and their complications including characteristics affections such as the regulation of vascular tone, vascular smooth muscle growth, monocyte adhesion, platelet function, and fibrinolytic activity, among others [136]. In terms of risk factors, the three world leading factors for cardiovascular diseases are (i) high systolic blood pressure (SBP), (ii) smoking, and (iii) high body mass index (BMI). Proper nutrition habits and healthy lifestyle play a major preventive role [134].

It has been widely reported that secoiridoids play a beneficial role against cardiovascular diseases based on their antioxidant and anti-inflammatory activities. Interesting studies performed with animal and cell models suggest that secoiridoids intake may be beneficial for the prevention and adjuvant treatment of such diseases (Table 3 and Table 4). Catalán et al. confirmed changes at proteomic level in cardiovascular tissues (aorta and heart tissues), down-regulating proteins related to the proliferation and migration of endothelial cells and occlusion of blood vessels in the aorta, and proteins related to heart failure in heart tissue in Wistar rats fed a secoiridoids-enriched diet [137].

One of the best-documented cardiovascular protector secoiridoids is OL. OL inhibited in a dose-dependent manner the copper sulfate-induced oxidation of LDL, reducing the formation of lipid peroxides and malondhial dehydelysine and 4-hydroxynonenol-lysine adducts [138]. These data indicated the protection of the apoprotein layer. Additionally, OL was able to scavenge superoxide anions generated by either polymorphonuclear cells or by the xanthine/xanthine oxidase system [139]. In this line, pretreatment with 20 μg/g of OL before ischemia–reperfusion in isolated rats hearts resulted in a significant reduction in creatine kinase and glutathione release, supporting experimental evidences of a direct cardioprotective effect of OL [145]. More recently, Parzonko and colleagues described that OL-treated endothelial progenitors cells (type CD31+ and VEGFR-2^+^) showed a decrease in the percentage of senescent cells and ROS formation and restored migration, adhesion, and tube formation. This effect was related to nuclear factor E2-related factor 2 (Nrf2) and heme oxigenase-1 (HO-1) expressions [140] (Table 3).

Relating to animal models, Andreadou and co-workers have developed several in vivo experimental models with OL treatment, defining the potential effect of this secoiridoid as a cardioprotector. Ischemia-treated rabbits fed with 10 or 20 mg/Kg/day OL-supplemented diets showed a reduction in infarct size, total cholesterol, and triglyceride concentrations [146]. Similarly, OL administrated via intravenous decreased some markers of cardiovascular disease in DOX-induced acute cardiotoxic rats such as creatine phosphokinase (CPK), lactate deshydrogenase, aspartate and alanine aminotransferase, and lipid peroxidation in myocardial tissue [147]. Completing this study, these authors revealed that OL down-regulated acetate and succinate levels and restored metabolic changes to normal levels in myocardial tissue [148]. Finally, Andreadou et al. concluded that OL administration could also prevent cardiomyopathy [149] (Table 4).

Concerning studies of atherosclerosis, OL could decreased serum lipids and tumor necrosis factor alpha (TNF-α) levels, which was accompanied by a down-regulation of monocyte chemostactic protein-1 and vascular cell adhesion molecule [150]. Ebaid et al. studied the effects of OL intake in obesity-induced cardiac metabolic changes. They found that an OL diet showed an increase of oxygen consumption, fat oxidation, and myocardial β-hydroxyacyl coenzyme A dehydrogenase activity and a reduction in the levels of lipid hydroperoxide and up-regulation of antioxidant enzyme confirmed that OL improved myocardial oxidative stress in standard-fed conditions [151].

With regard to OL antihypertensive effects, there are several studies performing different animal models. In this line, diabetic and hypetensive rats receiving 20, 40, or 60 mg/Kg/day of OL presented significantly reduced blood pressure, blood glucose, serum total cholesterol, LDL, triglyceride, MDA, coronary effluent creatine kinase, and coronary resistance. The animals also had high-density lipoprotein (HDL), erythrocyte SOD, left ventricular develop pressure, rate of rise, and rate of decrease of ventricular pressure [152,153,154]. Antihypertensive activity has also been supported by Ivanov et al., who reported significant changes in carotid and renal hemodynamics, reducing cardiovascular risk and improving vascular resistance in spontaneous hypertensive rat oxidative stress [155].

Regarding lipid regulation, the administration of OL and its aglycone form significantly down-regulated the serum levels of total cholesterol, triglycerides, and LDL and up-regulated HDL and liver antioxidant enzymes in Wistar rats fed a cholesterol-rich diet. These results demonstrated that secoiridoids administration could control the lipid peroxidation process, enhancing antioxidant enzyme activity [156].

The clinical trials of the effects of secoiridoids on cardiovascular diseases are scant. In this regard, 232 hypertension patients were involved in a clinical study subjected to a 500-mg oral dose of an OL-enriched extract administration twice daily for 8 weeks. The patients presented a significant reduction of systolic and diastolic blood pressure as well as the levels of triglycerides and LDL [159,160] (Table 4). Stock and colleagues measured cholesterol efflux capacity from free cholesterol-enriched macrophages to apolipoprotein B-depleted serum as the cholesterol acceptor in patients with coronary artery disease. OL showed a positive behavior against LDL oxidation, promoting cholesterol efflux and suggesting preventive effects against coronary artery diseases and enhanced atheroprotective actions [161].

According to OL aglycone, only few studies have been carried out. Dell’agli et al. described that OL aglycone exerted a modulation in early atherogenesis, reducing cell surface expressions of intracellular and vascular cell adhesion molecules (ICAM-1 and VCAM-1) in human umbilical vascular endothelial cells [141]. Similar to OL, the aglycone form was studied in rats fed with a cholesterol-rich diet by Jemai el at. The results suggested that the hypocholesterolemic effect of OL-aglycone might be due to its abilities to lower serum total cholesterol, triglycerides, and LDL cholesterol levels, slowing the lipid peroxidation process and enhancing SOD and CAT antioxidant enzyme activities, exhibiting a cardioprotective role against lipid oxidation and cholesterol efflux [156].

More recently, Leri et al. reported that OL-aglycone was able to reduce transthyretin toxicity in mouse atrial myocytes, so it could be used as treatment for severe cardiac symptoms [142]. In addition, Miceli and coworkers explored the effects of OL-aglyone in myocytes with an overexpression of monoamine oxidase-A (MAO-A), which is an enzyme that causes oxidative stress, autophagy flux blockade, and cell necrosis as a model of cardiac stress characterized by autophagy dysfunction. They observed that OL-aglycone counteracted the cytotoxic MAO-A effects [157]. Margheri et al. also reported the effects of OL aglycone on capillary morphogenesis induced by MRC5 fibroblast "senescense associated secretory phenotype" and progenitor endothelial cells, establishing that this secoiridoid could modulate angiogenesis indirectly on senescent fibroblasts [158] (Table 4). 

To date, OLE cardioprotective activity has been slightly investigated. Even so, some authors defend its cardioprotective property based on its capacity to inhibit COX-1 and COX-2 expression. It is well-known that thrombotic and cardiovascular disorders are linked to an imbalance in prostanoid homeostasis, particularly prostaglandin or thromboxane production, which are involved in vasodilatation or vasoconstriction, respectively, and platelet aggregation [162]. OLE has exerted strong inhibitory effects on COX-1 and COX-2 in several studies [50,163,164]; nevertheless, future studies are needed to confirm the property of OLE in cardiovascular disorders (Table 3).

The cardiovascular protection effects of OLA were tested in vitro in human neutrophils and monocytes. This compound was able to scavenge O_2_^−^, H_2_O_2_, and NO levels, among other parameters, which are implicated in tissue injury and chronic diseases, as atherosclerosis [32,165]. In a similar work, Czerwinska et al. studied the capacity of OLA on neutral endopeptidase (NEP) activity and other functions of human neutrophils, such as elastase, MMP-9 and interleukin (IL)-8 production, which was markedly increased in patients with myocardial infarction [32]. The authors concluded that OLA could play a role in the cardiovascular protective effects described by olive oil by inhibiting NEP activity, adhesion molecules expression, and elastase release. Likewise, Filipek and colleagues showed that OLA increased CD163 expression in human macrophages, supporting the significant role in attenuation of plaque destabilization induced by hemorrhages [144]. Later, these authors reported the beneficial effects of OLA in attenuating the destabilization of carotid plaque in 20 patients with hypertension. This work revealed the ability of OLA to modulate IL-10, HO-1, MMP-9, and high mobility group protein-1, which is a specific biomarker of cell lethality [166]. Thus, this compound could be potentially useful in the reduction of ischemic stroke risk. Concluding, the preventive and curative role of OLA, in terms of cardiovascular injury, could be attributed to its ability to regulate LDL oxidation and MPO activity, to reduce the expression of adhesion molecules, as angiotensin II production, and to confer protection to erythrocytes from oxidative hemolysis [33,73].

### 2.3. Olive Tree Secoiridoids and Neurodegeneration

Neurodegeneration is a process that leads to a progressive loss of structure or function of neurons, irreversible neuronal damage, death, and a common final pathway present in aging and neurodegenerative diseases. In addition, oxidative stress induced by impaired mitochondrial functions has been also reported [167]. Particularly, superoxide anion formation and the production of hydrogen peroxide are triggered by the induction of NADPH oxidase (NOX) subunit. This condition together with a high NO level, produced by the induction of inducible nitric oxide synthase (iNOS) results in the formation of peroxynitrite and nitrative stress [69]. Examples of neurodegenerative diseases include Alzheimer’s disease (AD), Parkinson’s disease (PD), Huntington’s disease, amyotrophic lateral sclerosis, frontotemporal dementia, and the spinocerebellar ataxias [168]. These diseases represent a primary health problem, especially in the aging population. Powerful experimental model organisms such as the mouse, fruit fly, nematode worm, and even baker’s yeast have been used for many years to explore neurodegenerative diseases and have provided key insights into these brain disorders.

Several epidemiological and observational studies support the belief that traditional alimentary regimens such as the Mediterranean diet where olive oil is the primary source of added fat is associated with improved aging and a reduced incidence of age-related diseases, including cardiovascular diseases, cancer, and cognitive decline [169]. Particularly, olive leaves and EVOO contain many functional phenolics that have been demonstrated to be able to reduce risk and offer protection against several aging and lifestyle-related diseases, including neurodegeneration, in both animal and human’s models. In fact, EVOO consumption has well-documented antioxidant, anti-inflammatory, anti-proliferative, anti-carcinogenic, and antibacterial effects [170]. Among the 200 different chemical compounds detected in olive oil, quantitatively, the class of secoiridoids is the most abundant. A number of different studies investigated the effects of secoiridoids from olive trees in both in vitro and in vivo models of AD and PD (Table 5 and Table 6).

AD is characterized by the increased accumulation of intracellular neurofibrillary tangles (NFTs) of hyperphosphorylated tau protein and of extracellular Aβ protein deposits (Aβ plaques) derived from amyloid precursor protein (APP) cleavage by γ-secretase and β-secretase. Dietary supplementation of OL (50 mg/Kg of diet) strongly improved the cognitive performance of young/middle-aged/aged TgCRND8 mice, and it also reduced ß-amyloid levels and plaque deposits. Moreover, OL-aglycone-fed mice brain displayed an astonishingly intense autophagy reaction [175,176,179]. Similar results were described in transgenic mice (APPswe/PS1dE9), where OL treatment showed significantly reduced amyloid plaque deposition in the cortex and hippocampus as compared to control mice [174]. Moreover, OL hindered the amyloid aggregation of Aβ (1-42) and its cytotoxicity and eliminated the appearance of early toxic oligomers, favoring the formation of stable harmless protofibrils, which were structurally different from the typical Aβ (1-42) fibrils [178]. Using transgenic CL2006 and CL4176 strains of *C. elegans* strains expressing Aβ42, as a simplified invertebrate model of AD, Diomede et al. evidenced that 50–100 µM OL-fed CL2006 worms displayed reduced Aβ plaque deposition, less abundant toxic Aβ oligomers, remarkably decreased paralysis, and increased lifespan with respect to untreated animals. A protective effect was also observed in CL4176 worms but only when OL was administered before the induction of the Aβ transgene expression [177] (Table 6).

In vitro studies have revealed that OL prevented the growth of toxic Aβ1-42 oligomers and blocked their successive growth into mature fibrils following its interaction with the peptide N-terminus and attenuated SH-SY5Y cell death caused by Aβ42, copper-Aβ42, and laevodihydroxyphenylalanine (l-DOPA)-Aβ42-induced toxicity after 24 h treatment, and a marked attenuated Aβ-induced astrocytes and microglia reaction was also found in the nucleus basalis magnocellularis (NBM) from adult male Wistar rats injected with Aβ42 aggregated with OL [171,174,177] (Table 5).

The potential protective effect of OLE in AD has been also investigated in TgSwDI mice. Mice treated for 4 weeks with OLE significantly decreased amyloid load in the hippocampal parenchyma and microvessels. This reduction was associated with enhanced cerebral clearance of Aβ across the blood–brain barrier (BBB), which was accompanied by an increase of P-glycoprotein (P-gp) and low density lipoprotein receptor-related protein 1 (LRP1) expressions, and activated the ApoE-dependent amyloid clearance pathway in the mice brains. The anti-inflammatory effect of OLE in the brains of these mice was also obvious where it was able to reduce astrocytes activation and IL-1β levels [181]. Similarly, 10 mg/kg of OLE administrated twice daily from 7 to 8 weeks of age and continued for 2 weeks (i.p.) enhanced the clearance of Aβ from C57BL/6 wild-type male mice brain and significantly increased the expression of P-gp and LRP1 [182]. In mouse brain endothelial cells (bEnd3), 25 and 50 µM OLE treatment resulted in a significant increase in P-gp and LRP1 levels [182]. Moreover, in a 5xFAD mouse model of AD, OLE-rich EVOO consumption, in combination with donepezil, significantly reduced Aβ load and related pathological changes, up-regulated synaptic proteins, enhanced BBB tightness, and reduced neuroinflammation associated with Aβ pathology [180] (Table 5 and Table 6).

PD is characterized by a progressive loss of dopaminergic neurons in the midbrain region known as *substantia nigra pars compacta* and by the presence of cytoplasmic protein aggregates called the Lewy body as well as Lewy neurites in remaining neurons.

Previous studies showed that OL inhibited αSN amyloidogenesis by directing αSN monomers into small αSN oligomers with lower toxicity, thereby suppressing the subsequent fibril growth phase [183]. The neuroprotective effect of OL has been explored in PC12 cells exposed to the potent parkinsonian toxin 6-hydroxydopamine (6-OHDA). OL treatment significantly decreased neuronal death and reduced the mitochondrial production of ROS resulting from blocking superoxide dismutase activity. Moreover, the quantification of autophagy and acidic vesicles in the cytoplasm alongside the expression of specific autophagy markers uncovered a regulatory role for OL against autophagy flux impairment induced by bafilomycin A1 [171,172].

### 2.4. Olive Tree Secoiridoids and Ageing

Aging is a natural biological process that involves the gradual decline of physiological function and the eventual failure of organism homeostasis followed by death. Aging is the process of accumulation of damages to cells, tissues, and organs of an individual that is universal and unique, thereby reducing the overall health of the organism. It is evident that aging can induce stress inside the system in the form of ROS or other stressors, reduce overall health, and induce age-associated neurological diseases [184]. The maintenance of homeostasis between the formation and elimination of damaged proteins is a key process in the development and growth of organisms [185]. To date, very few studies concerning the anti-aging effects of secoiridoids have been performed. However, secoiridoids suggest a potential age-related damage regulation based on their antioxidant, anti-inflammatory, and neuroprotector effects (Table 7 and Table 8).

Several in vitro studies have supported the potential of OL on the proteasome, which regulates the balance of cellular viability and is crucial in stress, aging, or senescent conditions [185]. The treatment of cell lysates from human embryonic fibroblast IMR90 enhanced three major proteasome catalytic activities: the chymo-trypsin-like (ch-L), the peptidylglutamyl-peptide hydrolase (PGPH) activity, and the trypsin-like (T-L). This activity was supported by Katsiki et al. OL-treated cells retained proteasome function during replicative senescence, and human embryonic fibroblast cultures exhibited a delay appearance of senescence morphology [186]. Santiago-Mora et al. reported the effects of OL on osteoblastogenesis and adipogenesis in mesenchymal stem cells from human bone marrow. OL stimulated osteoclastogenesis rising cellular matrix mineralization and inhibited bone desorption [187] (Table 7).

In terms of epigenetic, it has been postulated that oxidative damage to mitochondrial DNA (mtDNA) is one of several signs of age-related physiological consequences [188]. Fabiani and colleagues reported that OL and OL-algycone form counteracted DNA alterations in HL60 cells and peripheral blood mononuclear cell (PMBC) H_2_O_2_-induced DNA damage [43]. Moreover, OL counteracted bone loss and reduced α-1-acid glycoprotein plasma concentrations in senile osteoporosis rats [189]. Nikou et al. studied the effects of OLE and OLA in *Drosophila* flies, reporting that dietary administration of both of them was able to increase the T-L proteasome activity and 20S and 19S proteosomal subunits expression, leading to a significant reduction of ROS levels. Subsequently, it was reported that OLA up-regulated the gene expression of the proteasome, antioxidant response, and molecular chaperones in human skin fibroblasts [190] (Table 8). These data confirmed a potential anti-aging of both secoiridoids and suggested that these compounds could be critical nutraceuticals as preventive and therapeutic treatment of different age diseases. Nevertheless, clinical studies that confirm these suggestions need to be developed in the future.

### 2.5. Secoiridoids Olive Tree in Autoimmune Diseases

Autoimmune diseases are heterogeneous groups of diseases whose condition is that your immune system mistakenly attacks your body. In the normal state, the immune system is able to differentiate between foreign cells, such as viruses and bacteria, and its own cells. However, in an autoimmune disease, the immune system recognizes our own cells as foreign cells and it releases proteins called autoantibodies that attack healthy cells. These types of diseases could appear at any stage of life with higher or lower severity. For example, there are types of autoimmune disease that target only one organ, such as type 1 diabetes mellitus (T1DM), which damages the pancreas, whereas there are other diseases, such as systemic lupus erythematosus (SLE), which affect the whole body.

The National Institutes of Health (NIH) estimates that up to 23.5 million Americans suffer from autoimmune disease and that the prevalence rose in 2019. For this reason, autoimmune diseases are recognized as a major health problem. Nowadays, researchers have identified 80–100 different autoimmune diseases and suspect at least 40 more diseases of having an autoimmune basis. These diseases are chronic, can be life-threatening, and are responsible for death in female children and women in all age groups up to 64 years old [191]. The most characteristic examples of these kinds of autoimmune diseases are rheumatoid arthritis (RA), SLE, Crohn’s disease, ulcerative colitis (UC), and T1DM.

RA can be defined as a chronic inflammatory disease with a systemic autoimmune component, and it is mainly characterized by aggressive synovial hyperplasia, synovitis, the progressive destruction of cartilage, and bone erosion with the painful swelling of small joints, fatigue, prolonged stiffness and fever caused by immune responses, and specific innate inflammatory processes [192]. Worldwide, the annual incidence of RA is approximately three cases per 10,000 people, and the prevalence rate is approximately 1% increasing with age and peaking between the ages of 35 and 50 years old. RA affects all populations, although it is much more prevalent in some groups (e.g., 5–6% in some Native American groups) and much less prevalent in others (e.g., black persons from the Caribbean region) [193].

SLE can be defined as a chronic inflammatory and autoimmune disease that can affect multiple organ systems, including skin, joints, kidneys, and the brain, among others [194]. SLE is characterized by a deposition of immune complexes, which are formed in large amounts as antinuclear antibodies bind to the abundant nuclear material in blood and tissues, along with disturbances in both innate and adaptive immunity and T-cell signaling. In addition, SLE is characterized by its clinical and pathogenic complexity, difficult diagnosis, and the high number of complications that can affect the patient’s quality of life [195]. There are worldwide differences in the incidence and prevalence of SLE that vary with sex, age, ethnicity, and time. The highest estimates of incidence and prevalence of SLE were in North America: 23.2/10000 persons/years and 24/10000, people respectively. The lowest incidences of SLE were reported in Africa and the Ukraine (0.3/10000 persons/years), and the lowest prevalence was observed in Northern Australia (0 cases in sample of 847 people). Women were more frequently affected than men for every age and ethnic group. Incidence peaked in middle adulthood and occurred later for men. People of black ethnicity had the highest incidence and prevalence of SLE, whereas those with white ethnicity had the lowest incidence and prevalence. This appeared to be an increasing trend of SLE prevalence with time [196].

Crohn’s disease and UC are important chronic inflammatory disorders of the gastrointestinal system that contribute to the inflammatory bowel conditions, such as diarrhea with or without blood, abdominal pain, fever, weight loss, inflammation, and ulcers. These diseases have uncertain etiology but can be associated with multifactorial conditions in terms of immunity, genetics, and non-immune conditions such as environmental factors [197]. The total number of new cases of Crohn’s disease diagnosed each year (incidence) was 10.7 per 100,000 people or approximately 33,000 new cases per year. The total number of new cases of UC diagnosed each year was 12.2 per 100,000 people or approximately 38,000 new cases per year [198].

T1DM is considered an autoimmune disease that results from the destruction of pancreatic β-cells and is mediated by the immune system. This is caused by an autoimmune reaction where the body’s defense system attacks the cells that produce insulin. As a result, the body produces very little or no insulin. Multiple genetic and environmental factors found in variable combinations in individual patients are involved in the development of T1DM. Genetic risk is defined by the presence of particular allele combinations, which in the major susceptibility locus (the HLA region) affect T-cell recognition and tolerance to foreign and autologous molecules. T1DM can affect people at any age, but it usually develops in children or young adults. Around 10% of all people with diabetes have T1DM [199].

Due to the high prevalence and rising incidence of this kind of disease, nowadays, there is a requirement to investigate to develop palliative remedies or treatments that help us improve the symptoms and management of these types of diseases to improve the quality of life of patients. A new source of news alternative for autoimmune diseases is based on the use of natural compounds obtained from natural resources, such as *Olea europaea* L., which is traditionally used as diuretic, hypotensive, emollient, laxative, febrifuge, skin cleanser, and it is also used for the treatment of urinary infections, gallstones, bronchial asthma, and diarrhea. The published studies related to the effects of secoiridoids from the olive tree in these autoimmune diseases are summarized in Table 9 and Table 10.

## 3. Conclusions

There is evidence indicating that secoiridoids from the olive tree has a large potential as a therapy for a wide variety of ROS-related diseases. Interesting studies performed with animal and cell models suggest that secoiridods intake may be beneficial for the prevention and adjuvant treatment of such diseases. Particularly, dietary supplementation of OL, OL-aglycone, or OLE strongly improved the cognitive performance as well as reduced β-amyloid levels and plaque deposits favoring the formation of stable harmless protofibrils in transgenic mice models of AD. Likewise, using transgenic strains of *C. elegans*, OL-fed CL2006 worms displayed reduced Aβ plaque deposition, less abundant toxic Aβ oligomers, remarkably decreased paralysis, and increased lifespan. Besides, OL prevented the growth of toxic Aβ1-42 oligomers and cell death in SH-SY5Y cells, increased P-gp and LRP1 levels in mouse brain endothelial cells, and a marked attenuated Aβ-induced astrocytes and microglia reaction was also described in the NBM of adult male Wistar rats injected with Aβ42 aggregated with OL. The neuroprotective effect of OL secoiridoid has been slightly explored in PD. OL treatment has been demonstrated to inhibit αSN amyloidogenesis, suppressing the subsequent fibril growth phase, decreasing neuronal death, and reducing the mitochondrial production of ROS resulting from blocking superoxide dismutase activity in PC12 cells exposed to 6-OHDA.

The best-documented cardiovascular protector secoiridoid is OL. The literature reviewed here validates that the treatment of cells and animal models with OL could be beneficial in combating oxidative stress and thereby protect individuals from cardiovascular diseases. The beneficial effects of secoiridoids have been attributed to their antioxidant capacity and their ability to modulate cellular antioxidant defense mechanisms. In addition, OL has been shown to modulate a variety of targets, which include iNOS and NO, TNFα, IL-8 and MMP-2 and MMP-9 in addition to VCAM-1 and ICAM-1, and modulating signaling pathways by altering MAPK, NFκB, and Nrf2/HO-1, among others. Finally, secoiridoid supplemented diets exerted a reduction in infarct size, total cholesterol, and triglyceride concentrations. On the contrary, the cardiovascular protection effects of OLE and OLA as well as clinical trials of the effects of these secoiridoids on cardiovascular diseases are very scant, and future studies are needed to confirm them in cardiovascular disorders.

OL has also shown considerable anti-cancer effects against many types of cancer, including breast cancer, colorectal cancer, prostate cancer, pancreatic cancer, cervical carcinoma, and thyroid cancer both in vitro and in vivo. OL is believed to exert its anticancer activity via multiple mechanisms, interfering with different cellular pathways and inducing/inhibiting the production of various types of cytokines, enzymes, or growth factors such as MAPKs, NF-κB, Akt, COX-2, and STAT3. On the contrary, the studies reporting the anti-cancer effects of OLE and OLA are very circumscribed to breast cancer, hepatocellular carcinoma, and hematologic neoplasias.

On the other hand, the remarkable anti-inflammatory and immunomodulatory effects of these bioactive compounds have been reported in several in vitro and experimental models of RA, SLE, inflammatory bowel disease (IBD), T1DM, and multiple sclerosis (MS). Particularly, OL, OLA, and OLE may exert a remarkable inmmunodulatory and anti-inflammatory effects reducing the induced inflammatory response in murine macrophages and human fibroblasts. This was accompanied by amelioration of the production of essential pro-inflammatory cytokines involved in the regulation of the immune system response through the prevention of MAPKs and NF-κB pathways activation. Moreover, OL and OLA secoiridoids were effective in preventing the induced immuno-inflammatory response in animal experimental models of UC, MS, T1DM, and SLE. Finally, very few studies about secoiridoids’ anti-aging effects have been performed to date. Nevertheless, secoiridoids suggest posing a potential age-related damage regulation based on their antioxidant, anti-inflammatory, and neuroprotector effects.

In conclusion, the published data revealed, in general, consistent and very satisfactory results; however, the knowledge is very limited, especially in clinical trials. In this sense, further efforts are needed to mechanistically clarify the underlying biochemical and biological activities and pharmacokinetics/pharmacodynamics of secoiridoids from the olive tree in additional preclinical and clinical studies of ROS-related diseases.

## Figures and Tables

**Table 1:** Most recent in vitro studies that corroborate the important role of secoiridoids from the olive tree in the control and progression of different types of cancer.

Phenolic Compound	Cell Line	Concentration	Effects	Reference
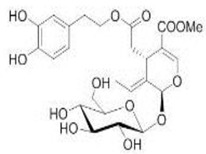 **OL**	NL-Fib: normal human skin fibroblasts; LN-18: poorly differentiated glioblastoma; TF-1a: erythroleukemia; 786O: renal cell adenocarcinoma; T-47D: infiltrating ductal carcinoma of breast-pleural effusion; MCF-7: human breast cancer; RPMI-7951: malignant melanoma skin-lymphoide metastasis; LoVo: colorectal adenocarcinoma-supraclavicular region metastasis	0.005%, 0.01% and 0.025% of OL in fibroblast tissue culture medium	OL inhibited cell growth, motility and invasiveness	[79]
	HT29 and SW260 human colon adenocarcinoma cell line	[0–100 µM]	OL might induce anti-proliferative and pro-apoptotic effects	[80]
	HT29	200, 400, and 800 µM	OL limited the growth and induced apoptosis via the p53 pathway	[81]
	MDA-MB-231 human breast cancer cell line	200 µg/mL	OL produced the up-regulating of TIMPs gene expression and the down-regulation MMPs overexpression gene	[82]
	MDA-MB-231; MCF-10A and MCF-7 human breast cancer cell lines	[0–300 µM]	OL exhibited specific cytoxicity against breast cancer cells, which is probably mediated through the induction of apoptosis via mitochondrial pathway	[83]
	SKBR3 breast cancer cell line	100 µM	OL worked as G-protein-coupled receptor (GPER) inverse agonists in estrogen receptor (ER)-negative and GPER-positive SKBR3	[84]
	MCF-7	100 and 200 µM	OL induced apoptosis in breast tumor cells via p53-dependent pathway	[85]
	MDA-MB-231 and MCF-7	[0–100 µM]	OL inhibited the viability of breast cancer cells and induced apoptosis via modulating NF-κB activation cascade	[86]
	MCF-7	[0–1200 µg/mL]	OL suppressed cells migration through suppression of epithelial-mesenchymal transition and could reduce DOX-induced side effects by reducing its effective dose	[87]
	MCF-7	[0–100 µM]	OL decreased the expression of both HDAC2 and HDAC3, induced apoptosis, and retarded cell migration and cell invasion in a dose-dependent manner	[88]
	MCF-7	200, 400, 600, and 1000 µM	OL inhibited the proliferation and invasion of cells by inducing apoptosis	[89]
	MDA-MB-231	[0–100 µM]	OL reduced cell viability in a dose-dependent manner; suppressed HGF or 3-MA, and induced cell migration and invasion	[90]
	MCF-7	[0–250 µM]	OL inhibited protein tyrosine phosphatase 1B (PTB1B)	[91]
	HepG2 and Huh7 human HCC cell lines	[0–100 µM]	OL induced apoptosis in HCC cells via the suppression of PI3K/Akt	[92]
	HepG2	100, 200 and 300 µM	OL could control the influencing of pro-nerve growth factor (NGF) and NGF balance via affecting MMP-7 activity without affecting the gene expression of NGF in HCC.	[93]
	LNCaP human prostate cancer androgen-responsive and DU145 androgen non-responsive cell lines	100 and 500 µM	OL reduced cell viability and induced thiol group modification	[94]
	TCP-1 and BCPAP thyroid tumor cell line	10, 50, and 100 µM	OL was able to inhibit in vitro thyroid cancer cell proliferation acting on the growth-promoting signal pathway	[95]
	HeLa human cervical carcinoma cell line	150 and 200 µM	OL-induced apoptosis was activated by the JNK/SPAK signal pathway	[96]
	SH-SY5Y human neuroblastoma cell line	350 µM	OL caused cell cycle arrest by down-regulating CyclinD1, CyclinD2, CyclingD3, CDK4, and CDK6 and up-regulating p53 and CDKN2A, CDKN2B, CDKN1A gene expressions. OL also induced apoptosis	[97]
	U251 and A172 human glioma cancer cell lines	0, 200, and 400 µM	OL inhibited cell viability and reduced the expression levels of MMP-2 and MMP-9. In addition, a specific PI3K inhibitor enhanced the pro-apoptotic and anti-invasive effects induced by OL	[98]
	HNE1 and HONE1 human nasopharyngeal carcinoma (NPC) cell lines	0 and 200 µM	OL treatments reduced the activity of the HIF-1α-miR-519d-PDRG1 pathway, which is essential to the radio-sensitizing effect of OL	[99]
	A549 human non-small cell lung cancer (NSCLC)	[0–200 µM]	OL caused a decrease in mithocondrial membrane potential, increase in Bax/Bcl2 ratio, release of mithocondrial cytochrome C, and activation of caspase 9 and caspase 3	[100]
	H1299 lung cancer cell line	[0–200 µM]	OL-induced apoptosis via the mitochondrial apoptotic cascade was activated by the p38 MAPK signaling pathway in H1299 cells	[101]
	A549 and BEAS-2B human noncancerous cell line	50 and 150 µM	OL induced apoptosis in A549 cells	[102]
	MIA PaCa-2, BxPC-3, and CFPAC-1 pancreatic cancer and HPDE non-tumorigenic pancreas cell lines	200 µM	OL arrested cell cycle, increased the Bax/Bcl-2 ratio, increased the activation of caspase 3/7, and induced apoptosis in MIA-PaCa-2	[103]
	A375 human melanoma cell line	[250–500 µM]	OL was able to stimulate apoptosis (500 µM), while at a dose of 250 µM it affected cell proliferation and induced the down-regulation of the pAkt/pS6 pathway	[104]
	OE-19 human esophagical cancer (EC) cell line	200 µM	OL inhibited the growth of EC cells as well as inhibiting HIF-1α and up-regulating BTG anti-proliferation F factor 3 (BTG3) expressions	[105]
	143B human osteosarcoma (OS) cell line	100 µM	OL showed alone and in combination with 2-methoxyestradiol a potent anti-cancer potential in highly metastatic OS cell	[106]
	AGS Human gastric adenocarcinoma cell line	[0–1000 µg/mL]	Magnetic nano-OL could trigger apoptosis in the AGS cell line	[107]
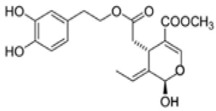 **OL-aglycone**	SH-SY5Y and RIN-5F insulinoma cell lines	100 µM	OL-aglycone triggered autophagy in cultured cells through the Ca2^+^-CAMKKβ–AMPK axis.	[108]
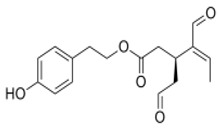 **OLE**	HT29 and HCT-116 human colon adenocarcinoma cell line	1, 2, 5, and 10 µg/mL	OLE produced an inhibition of AP1 activity and cyclooxygenase 2 (COX2) expression in HT29 cells	[109]
	MDA-MB-231, MCF-7, and PC3 prostate cancer cell lines	[0–20 µM]	OLE inhibited the proliferation, migration, and invasion of the epithelial human breast and prostate cancer cell lines and demonstrated anti-angiogenic activity	[110]
	BT-474, MDA-MB-231, and MCF-7	[0–60 µM]	OLE reduced the c-Met kinase activity, cell growth, migration, and invasion of breast cancer cells and induced G1 cell cycle arrest and apoptosis, as well as, inhibited c-Met-dependent signaling	[111]
	MDA-MB-231	[0–10 µM]	OLE showed strong anti-proliferative and down-regulated the expression of phosphorylated mTOR	[112]
	BT-474	[0–100 µg/mL]	OLE reduced breast cancer progression and locoregional recurrence models	[113]
	MDA-MB-231	5 mg/mL	OLE was able to control breast cancer progression	[114]
	BT-474 and MDA-MB-231	[0–200 µM]	OLE with the dual HER2/EGFR inhibitor, LP, induced synergistic tumor growth inhibition	[115]
	MCF-10A, MDA-MB-231, and MCF-7	1, 10, and 20 µM	OLE could be responsible for the selective activation of TRCP6-dependent Ca^2+^ influx and TRCP6 down-regulation at low µM concentrations	[116]
	Huh-7, HepG2, and HCCLM3 HCC cancer cell lines	[0–80 µM]	OLE inhibited proliferation and cell cycle progression and also inhibited HCC cell migration and invasion	[117]
	Huh-7, HepG2, and HCCLM3	5 and 10 µM	OLE reduced cell proliferation and increased cell death	[118]
	U937 hystocytic lymphoma cancer cell line	30 µM	OLE significantly inhibited the expression of Hsp90, a chaperone with a key role in cancer and neurodegeneration	[119]
	A375: A2058; HUVEC and HaCat cancer cell lines	[0–60 µM]	OLE suppressed STAT3 phosphorylation, decreased STAT3 nuclear localization, and inhibited STAT3 transcriptional activity	[120]
	Inmortalized human keratinocytes stimulated with epidermal growth factor	[0–100 µM]	OLE promoted the inhibition of ERK and Akt phosphorylation and the suppression of B-raf expression	[121]
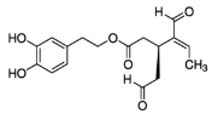 **OLA**	Inmortalized human keratinocytes stimulated with epidermal growth factor	[0–100 µM]	OLA promoted the inhibition of Erk and Akt phosphorylation and the suppression of B-raf expression	[121]
	HL60 human promyelocytic leukemia cell line	[0–10 µM]	OLA reduced the DNA damage at concentrations as low as 1 µM when co-incubated in the medium with H_2_O_2_	[43]
	NCI-H929; RPMI-8226; U266; MM1S and IIN3 human MM cancer cell lines	2.5, 5 and 10 µM	OLA elicited significant antitumor activity by promoting cell cycle arrest and apoptosis either with a simple agent or in combination with Carfilzomib	[122]

**Table 2:** Most recent in vivo studies that corroborate the important role of secoiridoids from the olive tree in the control and progression of different types of cancer.

Phenolic Compound	Animal Model	Doses	Effects	Reference
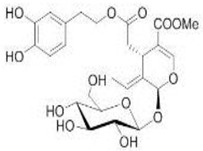 **OL**	Swiss albino with soft tissue sarcomas	1% OLE in drinking water	OL inhibited cell growth, motility, and invasiveness	[79]
	Male hairless mice (5 weeks old) were UVB irratied (36–180 mJ/cm^2^)	10 and 25 mg/Kg/day	OL increased the skin thickness and reductions in skin elasticity, skin carcinogenesis, and tumor growth	[123]
	DSS-induced CRC in C57BL/6 mice	50 and 100 mg/Kg	OL prevented the development of colonic neoplasia in by ameliorating colon inflammatory processes and limiting the activation of the main transcription factors involved	[124]
	Male Sprague–Dawley rats that received an injection of cisplatin (7 mg/Kg)	50, 100, and 200 mg/Kg/day	OL enhanced antioxidant activity and prevented oxidative stress, which it turn reduced 8-hydroxy-2’deoxy-guanosine (8-OH-dG) levels in lymphocytes of cisplatin-treated animals	[125]
	HNE1 and HONE1 injected into 6–8-week-old BalB/c mice	[0–200 µM]	OL was a radiation-sensitizing agent of NPC cells in an in vivo model	[99]
	Four-week-old C57BL/6N mice with HFD with or without OL and which were injected with B16F10 melanoma cells	0.02% and 0.04% enriched-diets	OL suppressed HFD-induced solid tumor growth and reduced HFD-induced expression of angiogenesis, lymphangiogenesis, and hypoxia markers	[126]
	Male Sprague–Dawley rats that received an injection of cisplatin (7 mg/Kg)	50, 100, and 200 mg/Kg/day	OL significantly decreased the formations of DNA damage and the level of malondialdehyde (MDA), and it increased the levels of total antioxidant status in pancreas tissue samples	[127]
	BalB/c OlaHsd-foxn1 injected with MDA-MB-231	50 mg/Kg	The combined treatment with OL and DOX downregulated the antiapoptosis and proliferation protein, nuclear transcription factor-kappa B (NF-κB), and its main oncogenic target Cyclin D1. It also inhibited the expression of Bcl-2	[128]
	Severe combined immunodeficiency mice (6 weeks-old) that received a subcutaneous injection of OE-19 cancer cells	200 µM	OL inhibited the growth of xenograft EC tumor as well as inhibited HIF-1α and upregulated B-cell translocation gene 3 (BTG3) expressions	[105]
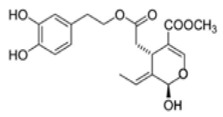 **OL-aglycone**	Transgenic hemizygous CRND8 mice harboring a double-mutant gene of APP695 and wild-type control lettermates with 4 and 10 months of age	100 µM	In OL-fed animals, there was a reduction of phospho-mTOR immunoreactivity and phosphorylated mTOR substrate p70 S6K levels	[108]
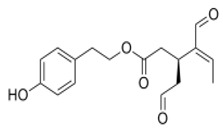 **OLE**	Swiss albino mice (6 weeks old)	10 mg/Kg/day	OLE reduced breast cancer progression and locoregional recurrence models	[113]
	Female athymic nude mice (Foxn1^nu^/Foxn1^+^) (4-5 weeks-old) inyected with BT-474 and MDa-MB-231	10 mg/Kg/day	OLE inhibited locoregional recurrence in luminal HER^2+^/ER^+^ BT-474 tumors	[129]
	Orthotopic tumor model of HCC in BalB/c mice	0, 5 and 10 mg/Kg/day	OLE suppressed tumor growth and impeded HCC metastasis in an in vivo lung metastasis model. OLE inhibited STAT3 activation and increased the activity of protein tyrosine hosphatase	[117]

**Table 3:** Beneficial effects of secoiridoids from the olive tree in the control and progression of different types of cardiovascular diseases: in vitro studies.

Phenolic Compound	Cell Line	Concentration	Effects	Reference
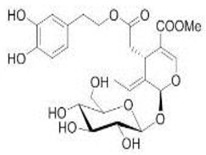 **OL**	Healthy human LDL	10 mM	OL inhibited LDL levels, lipid peroxides, malondial dehydelysine, 4-hydroxynonenal lysine adducts expression	[138]
	LPS-stimulates mouse macrophages		OL reduced superoxide anion generation, neutrophils respiratory burst, and hypochlorous acid	[139]
	Endothelial progenitors cells (CD31+ and VEGFR-2^+^)	[1–10 μM]	OL reduced senescent cells and reactive oxygen species (ROS) formation; restoration of migration, adhesion, tube formation, and the up-regulation of Nrf-2 and HO-1 expressions.	[140]
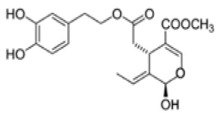 **OL-aglycone**	Human umbilical vascular endotelial cells	5 and 25 mM	OL-aglycone reduced cell surface expressions and mRNA levels of ICAM-1 and VCAM-1	[141]
	Mouse atrial myocites HL-1	60 mM	OL-aglycone inhibited tranthyretin toxicity	[142]
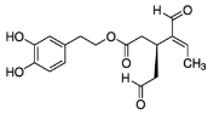 **OLA**	Human neutrophils and monocytes	[1–10 µM]	OLA proved to be stronger in the reduction of formyl-met-leu-phenylalanine and phorbol-myristate-acetate-induced oxidative bursts in neutrophils and myeloperoxidase release	[32]
	Human neutrophils	50 and 100 mM	OLA reduced elastase release, IL-8, MMP-9, and NEP activity	[143]
	Human macrophages	10–20 mM	OLA increased IL-10, HO-1, and CD163 expression	[144]

**Table 4:** Beneficial effects of secoiridoids from the olive tree in the control and progression of different types of cardiovascular diseases: in vivo and clinical studies.

In Vivo Studies				
Phenolic compound	Animal Model	Doses	Effects	Reference
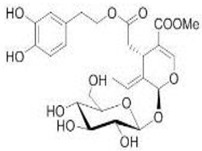 **OL**	Ischemia–reperfusion in insolated rat hearts	20 μg/g	OL reduced the creatine kinase, glutathione release, membrane lipid peroxidation	[145]
	Ischemic-treated hypercholesterolemic rabbits	10 or 20 mg/Kg/day	OL reduced the infarct size, total cholesterol, triglyceride concentration, and lipid peroxidation	[146]
	Doxorubicin-induced acute cardiotoxicity rats	100 or 200 mg/Kg	OL modulated the CPK, lactate deshydrogenase, aspartate and alanine aminotransferase, and lipid peroxidation	[147]
	Doxorubicin-induced acute cardiotoxicity in rats	100 or 200 mg/Kg	OL reduced the acetate and succinate levels. Restore metabolic changes	[148]
	Doxorubicin-induced chronic cardiomyopathy in rats	1000 or 2000 mg/Kg	OL controlled cardiac histopathology, nitro-oxidative stress, IL-6, myocardial metabolomics	[149]
	Rabbit model of atherosclerosis	100 mg/Kg	OL decreased lipids, cholesterol, LDL levels, TNF-α, NF-kB, ICAM-1, and VCAM-1 expressions	[150]
	Obesity-induced cardiac metabolic changes	0.023 mg/Kg/day	OL increased oxygen consumption, fat oxidation, and myocardial β-hydroxyacyl coenzyme A dehydrogenase activity and the up-regulation of antioxidant enzyme expression	[151]
	Renovascular hypertension and diabetes 2 rats	20, 40, or 60 mg/Kg/day	OL reduced blood pressure, blood glucose, serum total cholesterol, LDL, and triglycerides levels. Raised HDL levels.	[152]
	Diabetic hypertensive rats	20, 40, or 60 mg/Kg/day	OL lowered blood pressure, MDA, creatine kinase, and the induction of HDL levels	[153]
	Diabetic hypertensive rats	20, 40, or 60 mg/Kg/day	OL decreased blood pressure, glucose, and serum MDA levels. OL increased of HDL and erythrocyte SOD	[154]
	Spontaneous hypertensive rats	10 mg/Kg	OL reduced the oxidative stress, carotid and renal hemodynamics, blood pressure, and heart rate	[155]
	Rats fed with high-cholesterol diet	3 mg/Kg	OL modulated total cholesterol, triglycerides, LDL and HDL levels, and liver antioxidant enzymes	[156]
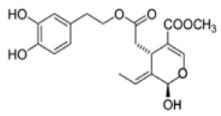 **OL-aglycone**	Neonatal rats ventricular myocytes with MAO-A enzyme overexpressed	100 μM	OL-aglycone decreased oxidative stress, autophagic flux blockade and cell necrosis	[157]
	Mature and progenitor endotelial cells	10 μM	OL-aglycone down-regulated NF-kB, IL-8, vascular endothelial growth factor (VEGF), MMP-2, and MMP-9	[158]
	Rats fed with high-cholesterol diet	3 mg/Kg	OL-aglycone modulated total cholesterol, triglycerides, LDL and HDL levels, and liver antioxidant enzymes	[156]
**Clinical Trials**				
**Phenolic Compound**	**Experimental System**	**Concentration**	**Effects**	**Reference**
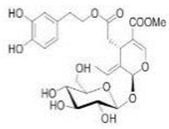 **OL**	232 hypertensive patients	500 mg twice daily	OL lowered systolic and diastolic blood pressure, triglycerides, and LDL levels	[159]

**Table 5:** In vitro studies that corroborate the effects of secoiridoids from the olive tree in different types of neurodegeneration processes.

Phenolic Compound	Cell Line	Concentration	Effects	Reference
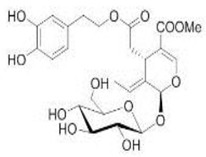 **OL**	6-OHDA-induced toxicity in rat adrenal pheochromocytoma (PC12) cells	20 and 25 μg/mL	OL decreased cell damage and reduce biochemical markers of PC12 cell death	[171]
	PC12 cells exposed to the potent parkinsonian toxin 6-OHDA	10 ^−12^ M	OL showed neuroprotective effects in an in vitro model of PD when administered preventively as a pretreatment. OL significantly decreased neuronal death. OL could also reduce the mitochondrial production of ROS resulting from blocking SOD activity	[172]
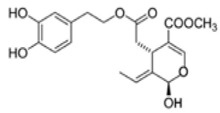 **OL-aglycone**	SH-SY5Y	[0–25 µM]	OL-aglycone prevented the growth of toxic Aβ1-42 oligomers and blocked their successive growth into mature fibrils following its interaction with the peptide N-terminus	[173]
	Exposure of SH-SY5Y cells with Aβ42	[10–1000 µM]	OL were able to attenuate cell death caused by Aβ42, copper-Aβ42, and [laevodihydroxyphenylalanine (l-DOPA)] l-DOPA-Aβ42-induced toxicity after 24 h	[174]
	NBM of adult male Wistar rats	450 µM	An apparent reduction in the amount of soluble A11-positive oligomers was detected in the NBM injected with Aβ42 aggregated with OL as compared with the NBM injected with Aβ42 alone	[175]
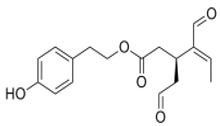 **OLE**	Mouse brain endothelial cells (bEnd3)	25 and 50 µM	Treatment of bEnd3 cells with OLE resulted in significant increase in P-gp and LRP1 levels	[173]

**Table 6:** In vivo studies that corroborate the effects of secoiridoids from the olive tree in different types of neurodegeneration processes.

Phenolic Compound.	Animal Model	Doses	Effects	Reference
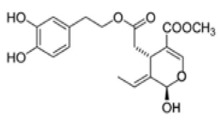 **OL-aglycone**	Double transgenic TgCRND8 mice, a model of amyloid-ß deposition	8 weeks dietary supplementation of OL-aglycone (50 mg/Kg of diet)	Dietary supplementation of OL-aglycone strongly improved the cognitive performance of young/middle-aged TgCRND8 mice, with respect to age-matched littermates with unsupplemented diet	[176]
	Transgenic mice (APPswe/PS1dE9)	50 mg/Kg of OL-aglycone containing olive leaf extracts (OLE) from 7 to 23 weeks of age.	Treatment mice (OL-aglycone) were showed significantly reduced amyloid plaque deposition (*p* < 0.001) in cortex and hippocampus in comparison	[174]
	Transgenic CL2006 and CL4176 strains of *C. elegans*	50 and 100 µM	OL-aglycone-fed CL2006 worms displayed reduced Aβ plaque deposition, less abundant toxic Aβ oligomers, remarkably decreased paralysis, and increased lifespan	[177]
	Systemic amyloidosis murine model	15 µM	OL-aglycone hindered amyloid aggregation of Aβ(1-42) and its cytotoxicity and eliminated the appearance of early toxic oligomers, favoring the formation of stable harmless protofibrils, which were structurally different from the typical Aβ(1-42) fibrils	[178]
	TgCRND8 mice	50 mg/Kg of diet during 8 weeks	OL-aglycone was active against glutaminylcyclase-catalyzed pE3-Aß generation, reducing enzyme expression and interfering both with Aß42 and pE3-Aß aggregation	[175]
	TgCRND8 (Tg) mice AD	Diet supplementation with OL-aglycone at 12.5 or 0.5 mg kg-1of diet	An OL-aglycone supplementation diet and the mix of polyphenols were found to improve significantly cognitive functions (*p* < 0.0001). Aß42 and pE-3Aß plaque area and number were significantly reduced in the cortex	[179]
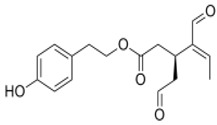 **OLE**	5xFAD mouse model of AD	EVOO rich with OLE	EVOO-rich OLE consumption in combination with donepezil significantly reduced Aβ load and related pathological changes	[180]
	TgSwDI mice	Daily i.p. with 5 mg/Kg OLE at 4 age of months and continued for 4 weeks.	OLE significantly decreased amyloid load in the hippocampal parenchyma and microvessels, which was associated with enhanced cerebral clearance of Aβ across the BBB	[181]
	C57BL/6 wild-type male mice	10 mg/Kg of OLE twice daily from 7 to 8 weeks of age andcontinued for 2 weeks (i.p.)	OLE enhanced clearance of Aβ from the brain. A significant increase in the expression of P-gp and LRP1 was also observed in the brain microvessels	[182]

**Table 7:** Potential role of secoiridoids obtained from the olive tree in anti-aging: in vitro studies.

Phenolic Compound	Cell Line	Concentration	Effects	Reference
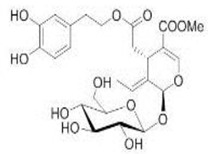 **OL**	Human embryonic fibroblast (IMR90)	[0.1–50 mM]	OL enhanced ch-L, PGPH, and PGPH proteasome activity	[185]
	Human embryonic fibroblast		OL retained proteasome function during replicative senescence and delayed in the appearance of senescence morphology	[186]
	Mesenchymal stem cells from human bone marrow	[1–100 µM]	OL enhanced osteogenic gene expression markers and osteoblast phenotypic characteristic	[187]
	Human promyelocitic leukemia cells (HL60)	10 μM	OL restored DNA damage	[43]
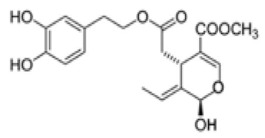 **OL-aglycone**	Human promyelocitic leukemia cells (HL60)	10 μM	OL-aglycone restored DNA damage	[43]
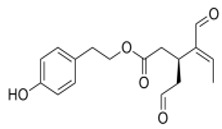 **OLE**	Normal human skin fibroblasts	50, 100, 150, and 200 μM	OLE up-regulated genes’ expression of proteasome, antioxidant responses, and molecular chaperones genes	[190]
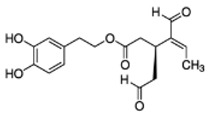 **OLA**	Normal human skin fibroblasts	50, 100, 150, and 200 μM	OLA up-regulated genes’ expression of proteasome, antioxidant responses, and molecular chaperones genes	[190]

**Table 8:** Potential role of secoiridoids obtained from the olive tree in anti-aging processes: in vivo studies.

Phenolic Compound	Animal Model	Doses	Effects	Reference
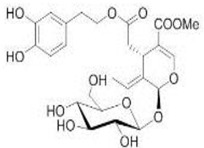 **OL**	Senile osteoporosis rats model	15 mg/kg	OL counteracted bone loss and reduce α-1-acid glycoprotein plasma concentration	[189]
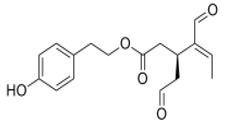 **OLE**	*Drosophila* in vivo model	Dietary supplementation of OLE: 400 nM, 200 nM, and 100nM.	OLE increased the ch-L proteasome activity and the expression of 20S and 19S proteasomal subunits and decreased of ROS levels in somatic tissues of *Drosophila* flies	[190]
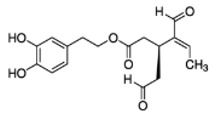 **OLA**	*Drosophila* in vivo model	Dietary supplementation of OLA: 400 nM, 200 nM, and 100 nM	OLA increased the ch-L proteasome activity and the expression of 20S and 19S proteasomal subunits and decreased of ROS levels in somatic tissues of *Drosophila* flies.	[190]

**Table 9:** Effective mechanisms and concentrations of bioactive secoiridoids from the olive tree in in vitro models of immunoinflammatory diseases.

Phenolic Compound	Cell Line	Concentration	Effects	Reference
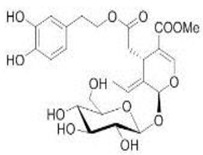 **OL**	LPS-stimulated murine peritoneal macrophages.	25 and 50 µM	OL reduced pro-inflammatory cytokines levels and interferon (IFN)-γ, as well as iNOS and COX-2 overexpressions	[200]
	Human synovial fibroblasts cell line (SW982)	50 and 100 µM	OL pre-treatment down-regulated mitogen active protein kinase (MAPK)s and NF-κB and induction of Nrf2-linked HO-1 signaling pathways	[201]
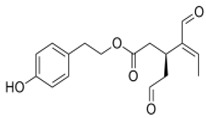 **OLE**	LPS-stimulated murine peritoneal macrophages.	[25–100 µM]	OLE showed a potent reduction of ROS, nitrites, and pro-inflammatory cytokines levels. OLE inhibited canonical and noncanonical inflammasome signaling pathways	[50]
	J774 LPS-stimulated macrophages	50 µM	OLE inhibited LPS-induced NO production without affecting cell viability	[117]

**Table 10:** Effective mechanisms and concentrations of bioactive secoiridoids from the olive tree in in vivo models and clinical trials of immunoinflammatory diseases.

In Vivo Studies				
Phenolic compound	Animal Model	Doses	Effects	Reference	
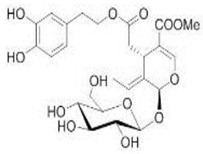 **OL**	Diabetes type I model induced by subcutaneous alloxan monohydrate injection in Sprague-Dawley male rats	Oral gavage 15 mg/Kg/day of OL	OL significantly decreased leucocyte infiltration and glomerulosclerosis. OL decreased the levels of urea, nitrite, and creatinine and decreased MPO activity	[202]	
	Experimental autoimmune myocarditis (EAM) model induced by porcine cardiac myosin in Lewis rats	Oral gavage 20 mg/Kg/day of OL	OL improved cardiac functions and attenuate inflammatory cell infiltration and cytokine expression levels	[203]	
	Chronic colitis model induced by DSS (1% in first and second cycles and 2% in third and fourth cycle) in female C57BL/6 mice (6–8 weeks at age weighting 18–20 g)	Diet supplemented with 0.25% OL	OL exhibited a decrease of inflammatory symptoms and decreased inflammatory cell recruitment	[204]	
	Acute colitis model induced by DSS (5%) for 7 days in BALB/c mice (6–8 weeks at age weigthing 18–20 g)	Diet supplemented with 1% OL	Oral administration of OL attenuated the extent and severity of acute colitis and reduced production of inflammatory mediators	[205]	
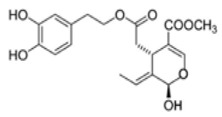 **OL-aglycone**	Collagen type II-induced arthritis (CIA) in three-week-old male DBA-J/1	Oral gavage 40 mg/Kg/day of OL	OL prevented joints inflammation and reduced inflammatory mediators overexpression and cytokines levels	[206]	
**Clinical Trials**					
**Phenolic Compound**	**Cells**	**Concentration**	**Effects**	**Reference**	
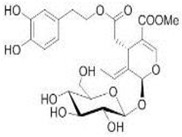 **OL**	14 outpatients biopsies with Ulcerative Colitis	3 µM OL from olive leaves from *Olea europaea* L.	OL reduced the expression of COX-2 and IL-17 in samples treated with OL. In addition, OL ameliorated inflammatory tissular damage	[207]	

## Abbreviations

3:4-DHPEA-EDA	3,4-(dihidrophenyl)etanol
3-MA	3-methyladenine
6-OHDA	6-hydroxydopamine
8-OH-dG	8-hydroxy-2’deoxy-guanosine
AD	Alzheimer’s disases
AGE	advanced glycoxidation products
Akt	Protein kinase B
ALE	Advanced lipid peroxidation products
APP	Amyloid precursor protein
ASM	acid sphingomyelinase
AUC	area under the curve
BBB	Blood-brain barrier
BMI	Body mass index
BMSC	Bone marrow stromal cells
BTG3	B-cell translocation gene 3
ch-L	Chymo-trypsin-like
CIA	Collagen-induced arthritis
c-MET	Tyrosine-protein kinase Met
COX	Cyclooxigenase
CPK	Creatine phosphokinase
CRC	Colorrectal cancer
DSS	Dextran sulfate sodium
EAM	Experimental autoimmune myocarditis
EC	Esophagical cancer
ER	Estrogen receptor
ERK	Extracellular signal-regulated kinases
EVOO	Extra virgin olive oil
GES	Geraniol synthase
GPER	G-protein coupled receptor
GPx	Glutathione peroxidase
GT	Glucosyltransferase
h	hours
HCC	Hepatocellular carcinoma
HDAC	Histone deacetylase
HDL	High-density lipoprotein
HFD	High fat diet
HGF	Hepatocyte growth factor
HIF-1α	Hypoxia-inducible factor 1α
HO-1	Heme oxigenase-1
HTy	Hydroxytyrosol
IBD	Inflammatory bowel disease
ICAM-1	Intracellular adhesión molecule-1
IL	Interleukin
iNOS	Inducible nitric oxide synthase
i.p.	intraperitoneally
JNK	c-Jun N-terminal kinase
LDL	Low-density lioprotein
LMP	Lyposomal membrane permeabilization
LP	Lapatinib
LRP1	Lipoprotein receptor-related protein 1
MAO-A	Monoamine oxidase-A
MDA	Malodialdehyde
min	minutes
MIP-α	Macrophage inflammatory 1α
MM	Multiple mieloma
MMPs	Metalloproteinases
MPO	Myeloperoxidase
MS	Multiple sclerosis
mtDNA	mitochondrial DNA
mTOR	mammalian target of rapamycin
NADH	Nicotinamide adenine dinucleotide reduced form
NBM	Nucleus basalis magnocellularis
NEP	Neutral endopeptidase
NFTs	Neurofibrillary tangles
NF-κB	Nuclear transcription factor-kappa B
NGB	Pro-nerve growth factor
NIH	National institute of Health
NO	Nitric oxide
NOX	NADPH oxidase
NPC	Nasopharingeal carcinoma
Nrf2	Nuclear factor E2-related factor 2
NSCLC	Non-small cell lung cáncer
OL	Oleuropein
OLA	Olacein
OLE	Oleocanthal
OS	Osteosarcoma
PD	Parkinson’s disease
P-gp	P-glycoprotein
PGPH	Peptidylgutamyl-peptide hydrolase
PI3K	Phosphatidylinositol 3-kinase
PBMC	Peripheral blood mnonuclear cells
PTB1B	Protein tyrosine phosphatase 1B
RA	Rheumatoid arthritis
ROS	Reactive oxygen species
SBP	Systolic blood pressure
SLS	Secologanin synthase
SPAK	Ste20-like proline alanine rich kinase
SLE	Systemic lupus erythematosus
STAT	Signal transducer and activator of transcription
T1DM	Type 1 Diabetes mellitus
TIMPs	Tissue inhibitors of metalloproteinases
T-L	Trypsin-like
Ty	Tyrosol
UC	Ulcerative colitis
VCAM-1	Vascular cell adhesión molecule-1
VEGF	Vascular endotelial growth factor
